# Energy Expenditure in Critically Ill Adult Patients With Acute Brain Injury: Indirect Calorimetry vs. Predictive Equations

**DOI:** 10.3389/fneur.2019.01426

**Published:** 2020-01-23

**Authors:** Kathryn A. Morbitzer, William S. Wilson, Alex C. Chaben, Adrienne Darby, Kelly A. Dehne, Emily R. Brown, Denise H. Rhoney, J. Dedrick Jordan

**Affiliations:** ^1^Division of Practice Advancement and Clinical Education, UNC Eshelman School of Pharmacy, Chapel Hill, NC, United States; ^2^Department of Pharmacy, University of North Carolina Health Care, Chapel Hill, NC, United States; ^3^Department of Nutrition and Food Services, University of North Carolina Health Care, Chapel Hill, NC, United States; ^4^Departments of Neurology and Neurosurgery, University of North Carolina School of Medicine, Chapel Hill, NC, United States

**Keywords:** acute brain injury, critically ill, energy expenditure, indirect calorimetry, predictive equations

## Abstract

**Introduction:** Predictive equations (PE) are used in lieu of indirect calorimetry (IC) due to cost and limited resources; however, these equations may not be as accurate as IC in estimating resting energy expenditure (REE) in critically ill patients, putting them at risk of malnutrition. The purpose of this study is to compare predicted and measured energy expenditure (MEE) in critically ill adults with acute brain injury.

**Materials and Methods:** This was a retrospective review of adult patients admitted to the Neurosciences ICU with acute brain injury between May 1st, 2014 and April 1st, 2016 who had IC performed. The Harris Benedict (HBE), Penn State University, and Mifflin St Jeor (MSJ) PE were used in comparison to IC results. Subgroup analyses stratified patients based on BMI and type of acute brain injury.

**Results:** One hundred and forty-four patients met inclusion criteria. Comparing predicted and MEE found no significant difference (*p* = 0.1). High degrees of interpatient variability were discovered, with standard deviations ranging from 17 to 29% of each PE. Pearson's correlations indicated weak associations when HBE, Penn State, and MSJ were individually compared to MEE (*r* = 0.372, 0.409, and 0.372, respectively). A significant difference was found between predicted and MEE in patients with a BMI < 30 kg/m^2^ (*p* < 0.01) and in those with aneurysmal subarachnoid hemorrhage (*p* < 0.01).

**Discussion:** Due to interpatient variability that exists among REE of critically ill patients with acute brain injury, IC should be used when feasible.

## Introduction

Nutrition therapy in critically ill patients is an important component of their overall care, and if not appropriately considered can result in poor outcomes (1). Indirect calorimetry (IC) is a non-invasive method that measures resting energy expenditure (REE) and is the gold standard for predicting energy requirements in critically ill adult patients (1–7). IC measures REE by measuring whole-body oxygen and carbon dioxide gas exchange. This concept is based on the strong correlation between intake of oxygen and release of carbon dioxide with energy production. Although IC is the gold standard for measuring REE, cost and resources necessary for performing IC may be barriers for institutions to implement such services.

Predictive equations are often used to estimate REE. A variety of predictive equations are available and are known to be less accurate, with accuracy rates ranging from 40 to 75% when compared to IC, especially in critically ill patients (8–11). Predictive equations use various patient parameters including height, weight, sex, and age, as seen in Table 1, to estimate REE. No single equation has proven to be more accurate in the intensive care unit (ICU) setting (1, 12). Furthermore, REE for patients with acute brain injuries has been estimated to be between 40 and 200% above that of a non-injured person (7).

**Table 1:** Predictive equations used to estimate REE^*^.

**Predictive equation name**	**Equation**
Harris Benedict (HBE)	Male: [66.4730 + (13.7516 × weight) + (5.0033 × height) – (6.7550 × age)] × 1.2 Female: [655.0955 + (9.5634 × weight) + (1.8496 × height) – (4.6756 × age)] × 1.2
Penn State 2003	Male/Female: 0.85 × HBE + (175 x T_max_) + (33 x V_e_) − 6433
Mifflin St Jeor (MSJ)	Male: [(9.99 × weight) + (6.25 × height) – (4.92 × age) + 5] × 1.25 Female: [(9.99 × weight) + (6.25 × height) – (4.92 × age) – 161] × 1.25
ASPEN weight-based equations, average	Male/Female: (25 kcal/kg + 30 kcal/kg) / 2

*REE, resting energy expenditure; ASPEN, American Society for Parenteral and Enteral Nutrition; kcal, kilocalories*.

* *The HBE and MSJ equations were multiplied by correction factors of 1.2 and 1.25 to calculate REE, respectively; weight, admission weight in kilograms; height, admission height in centimeters; age, years; T_max_, maximum temperature (Celsius) 24 h prior to IC reading; V_e_, minute ventilation (L/min); ASPEN weight-based equations (25 kcal/kg and 30 kcal/kg) were calculated for each patient and averaged to obtain ASPEN average*.

Overfeeding and underfeeding critically ill patients has been shown to lead to poor outcomes and complications such as electrolyte imbalances, impaired organ function, and failure to wean mechanical ventilation, potentially leading to increasing length of stay and accruing additional costs to the patient and institution (10, 11, 13–15). Providing optimal caloric intake is critical for patients with acute brain injury, yet optimal caloric intake in these patients remains unclear (16). This study is designed to compare the predicted energy expenditure (PEE), as calculated by predictive equations, and measured energy expenditure (MEE) using IC in patients with acute brain injury to allow clinicians to better identify the most accurate assessment of nutrient requirements of these patients to provide best practices in nutrition support.

## Materials and Methods

This was a single-center, observational, retrospective study approved by the Institutional Review Board and included patients at least 18 years old, admitted to the Neurosciences Intensive Care Unit (NSICU) with acute brain injury between May 2014 and April 2016, with a completed IC measurement. Exclusion criteria included factors that could significantly alter IC results including, specifically, air leaks from chest or endotracheal tubes and in the ventilation circuit, fraction of inspired oxygen ≥0.6, respiratory quotient < 0.67 or >1.25, or if IC was performed while the patient was receiving any active renal replacement therapy, including hemodialysis, peritoneal dialysis, or continuous renal replacement therapy (CRRT).

Predictive equations used were the most commonly cited in the literature and included the Harris-Benedict equation modified for critically ill patients (HBE), 2003 Penn State University equation, Mifflin-St Jeor (MSJ) modified for patients with minimal activity equation, and a modified version of the American Society for Parenteral and Enteral Nutrition (ASPEN) weight-based equations (Table 1) (7, 17). This modified version was used to provide an average of the minimum and maximum kilocalories/kilogram (kcal/kg) range recommended for patients with a body mass index (BMI) < 30 kg/m^2^ in the ASPEN 2016 guidelines (7).

Patient characteristics at baseline were recorded. This included patient demographics (age, sex, race, height, and weight), type of brain injury, comorbid conditions, pregnancy status, admission sequential organ failure assessment (SOFA) score, admission Glasgow Coma Scale (GCS) score, and length of stay in the NSICU. Additional information regarding the patient's status during IC measurement were recorded including, renal replacement therapy, MEE from IC, date of completed IC, maximum temperature within 24-h prior to IC reading, temperature modulation (controlled normothermia, hypothermia, or none), respiratory quotient, minute volume, fraction of inspired oxygen, GCS, bedside shivering assessment scale (BSAS) score, Richmond agitation-sedation scale (RASS) score, infection (confirmed, suspected, or none), as well as whether or not the patient was receiving sedatives, paralytics, vasopressors, or a pentobarbital infusion. Presence of infection was considered confirmed if the patient was being treated with antimicrobials and if there was a positive culture or diagnostic scans indicating a source of infection; all others were deemed suspected or not infected.

For analyses consisting of all patients, continuous variables are represented as mean ± standard deviation (SD), ordinal variables represented as median (interquartile range, IQR), and categorical variables are represented as n (%). PEE and MEE were compared using ANOVA; the predictive equations included in this comparison were HBE, Penn State, and MSJ. The ASPEN equation was not included in this comparison as its recommended use is in patients with a BMI < 30 kg/m^2^, and all patients, regardless of BMI, were being included in this analysis (7). The correlation of individual PEE equations and MEE was determined using Pearson's correlation coefficient; the predictive equations included in these analyses were HBE, Penn State, MSJ, and ASPEN weight-based equation. In the ASPEN weight-based equation comparison with MEE, only patients with a BMI < 30 kg/m^2^ were included.

Subgroup analyses comparing PEE and MEE were performed in patients based on BMI and type of acute brain injury. In patients with a BMI < 30 kg/m^2^, PEE as estimated by HBE, Penn State, MSJ, and ASPEN were compared; in patients with BMI ≥ 30 kg/m^2^, PEE as estimated by HBE, Penn State, and MSJ were compared. In patients with aneurysmal subarachnoid hemorrhage (aSAH), intracranial hemorrhage (ICH), and traumatic brain injury (TBI), with a BMI < 30 kg/m^2^, PEE as estimated by HBE, Penn State, MSJ, and ASPEN were compared. In the subgroup analyses, comparisons between the PEE and MEE were performed using the Kruskal–Wallis test.

For all analyses, statistical significance was defined as a *p* < 0.05. The statistical tests for this study were performed using StataSE version 14 (StataCorp, College Station, TX).

## Results

There were 180 patients identified to have IC completed during the study period. Of these, 144 met inclusion criteria and were included in the final analyses. Table 2 summarizes patient baseline characteristics as well as patient status at the time of IC measurement. The average patient age was 55 years, 53.5% female, and 52.8% white, with an average BMI of 27.5 kg/m^2^. The average SOFA score upon admission to the NSICU was 5 with the most prevalent admission diagnosis of acute brain injury being aSAH (28.5%), ICH (27.1%), and TBI (15.3%).

**Table 2:** Patient demographics and characteristics.

**Variable**	**All patients (*n* = 144)**
Age, years (mean ± SD)	55.1 ± 16.9
Aneurysmal subarachnoid hemorrhage	55.4 ± 16.5
Intracerebral hemorrhage	55.2 ± 16.7
Traumatic brain injury	55 ± 16.9
Acute ischemic stroke	55.7 ± 16.4
Intracranial tumor	55.1 ± 16.4
Status epilepticus	54.8 ± 16.6
Other	54.9 ± 16.6
Female Gender, *n* (%)	77 (53.5)
Race, *n* (%)	
African American	47 (32.6)
White	76 (52.8)
Asian	2 (1.4)
Non-white Hispanic	4 (2.8)
American Indian	2 (1.4)
Unknown	13 (9)
Height, cm (mean ± SD)	171.2 ± 10.5
Weight, kg (mean ± SD)	80.5 ± 19.6
BMI, kg/m^2^ (mean ± SD, *n*%)	
Total	27.5 ± 6.7
BMI < 30	24.6 ± 3.6 (72.9)
BMI ≥ 30	35.5. ± 6.5 (27.1)
Type of brain injury, *n* (%)	
Aneurysmal subarachnoid hemorrhage	41 (28.5)
Intracerebral hemorrhage	39 (27.1)
Traumatic brain injury	22 (15.3)
Acute ischemic stroke	20 (13.9)
Intracranial tumor	8 (5.6)
Status epilepticus	8 (5.6)
Other	6 (4.2)
Admission SOFA score, (median [IQR])	5 [3–6.5]
Comorbidities, *n* (%)	
Hypertension	83 (57.6)
Hyperlipidemia	42 (29.2)
Diabetes	35 (24.3)
Patient status at time of first IC	
Temperature modulation, *n* (%)	
None	119 (82.6)
Controlled normothermia	24 (16.7)
Hypothermia	1 (0.7)
Sedation at time of IC, *n* (%)	91 (63.2)
Type of sedation, *n* (%)	
Infusion(s) only	71 (78.0)
Intermittent scheduled only	12 (13.2)
Combination of infusion + scheduled	8 (8.8)
Vasopressors at time of IC, *n* (%)	26 (18.1)
Infection at time of IC, *n* (%)	
None	58 (40.3)
Confirmed	59 (40.9)
Suspected	27 (18.8)
Days from admission to completed IC (median [IQR])	5 [2–9]

The median time from admission to the NSICU to the completion of IC was 5 days (IQR 2–9). At the time of the IC reading, the study median GCS and RASS scores were 7 (IQR 6–10) and −2 (IQR −4 to −1), respectively; additionally, all patients had a BSAS of 0 during their IC reading. Of the 144 patients, 91 were receiving sedation, 26 were receiving vasopressors, and 3 were receiving pentobarbital infusions. Most patients did not require thermoregulation (82.6%) and 59 (40.9%) patients had a confirmed infection during their first IC test; 27 (18.8%) had a suspected infection, and 58 (40.3%) patients did not have a documented infection (Table 2).

**Figure 1:**
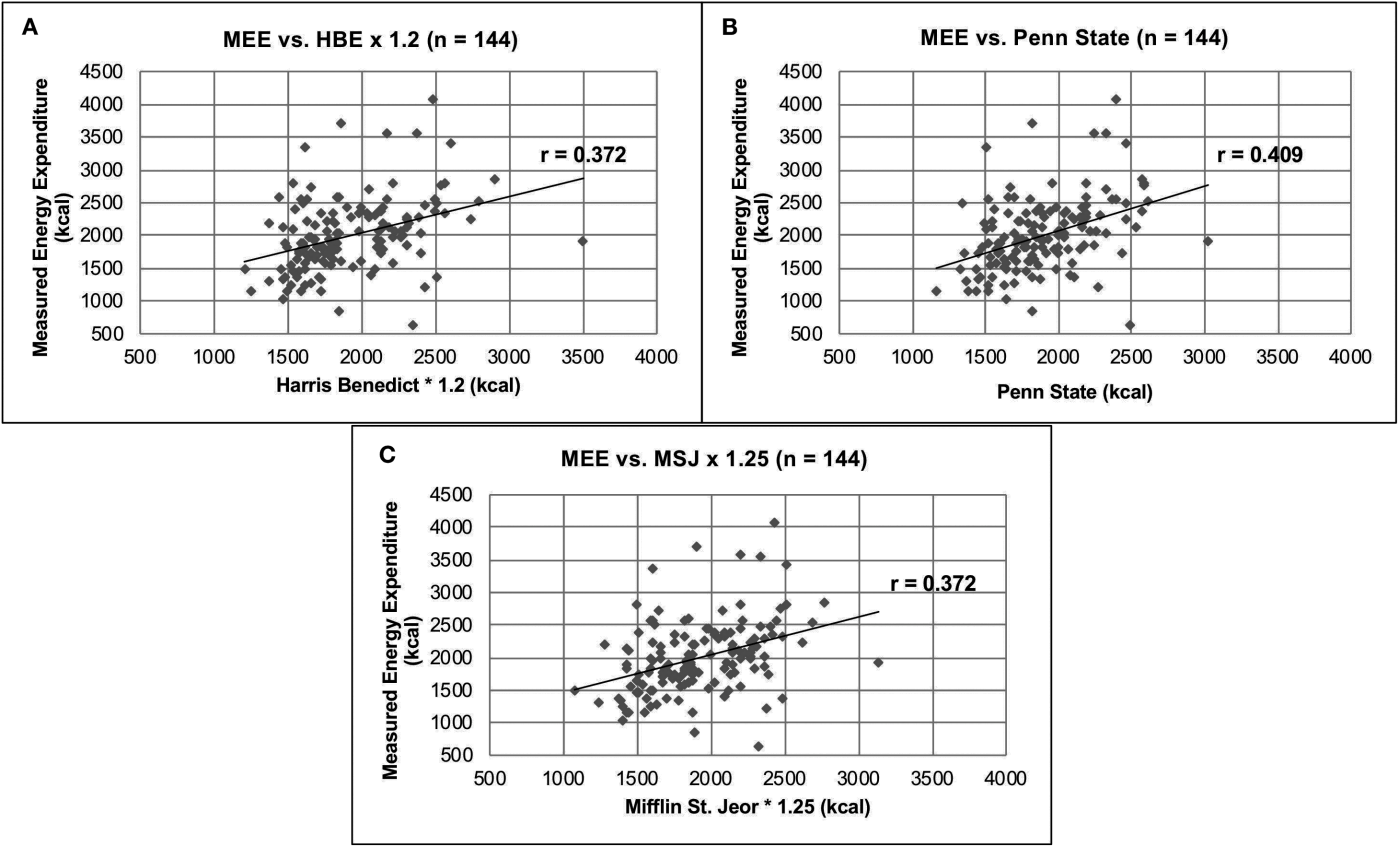
Comparison of MEE vs. PEE. **(A)** MEE vs. HBE × 1.2 (*n* = 144). **(B)** MEE vs. Penn State (*n* = 144). **(C)** MEE vs. MSJ × 1.25 (*n* = 144).

The mean MEE as determined by IC was 1,995 ± 554 kcals, compared to the PEE of 1,919 ± 373, 1,888 ± 333 and 1,914 ± 355 kcals as determined by the HBE, Penn State, and MSJ equations, respectively (*p* = 0.1) (Table 3). Although no significant difference was found, large standard deviations suggested wide interpatient variability. Pearson's correlation was completed in order to compare individual PEE equations and MEE. Of the three predictive equations (Figures 1A–C), the Penn State equation had the strongest correlation with MEE, despite a relatively low *r*-value (*r* = 0.409).

**Table 3:** Energy expenditure for all patients (*n* = 144).

**Variable**	**Energy expenditure mean ± SD (kcals)**	***P*-value**
MEE	1,995 ± 554	0.1
HBE × 1.2	1,919 ± 373	
Penn State 2003	1,888 ± 333	
MSJ x 1.25	1,914 ± 355	

**Figure 2:**
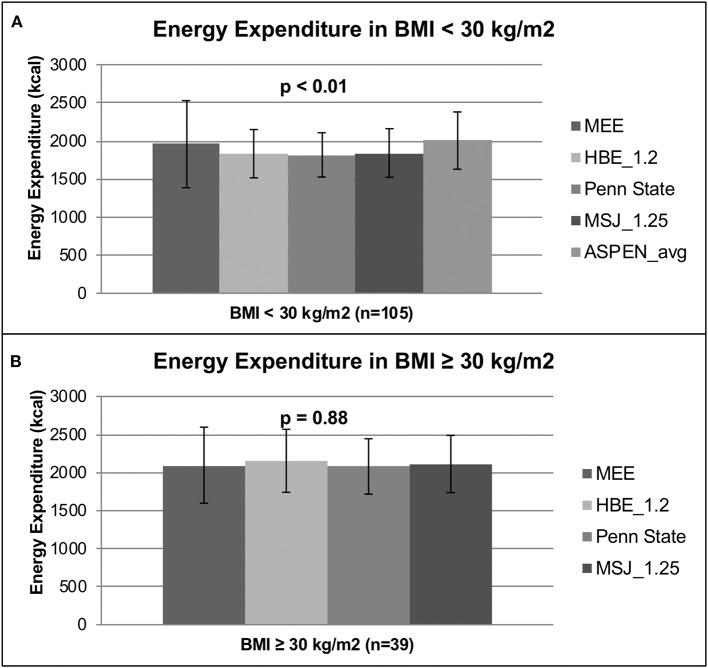
Energy expenditure based on BMI. **(A)** Energy expenditure based on BMI < 30 kg/m^2^. **(B)** Energy expenditure based on BMI ≥ 30 kg/m^2^.

In the subgroup analysis of patients with a BMI < 30 kg/m^2^ (*n* = 105), the mean MEE as measured by IC was 1,957 ± 571 kcal compared to the PEE of 1,832 ± 317, 1,816 ± 291, 1,841 ± 319, and 2,004 ± 376 kcals as predicted by the HBE, Penn State, MSJ, and ASPEN equations, respectively (*p* < 0.01) (Figure 2A). In the subgroup analysis of patients with a BMI ≥ 30 kg/m^2^ (*n* = 39), the MEE as determined by IC was 2,096 ± 500 kcals compared to the PEE of 2,154 ± 414, 2,080 ± 364, and 2,112 ± 376 kcal as estimated by the HBE, Penn State and MSJ equations, respectively (*p* = 0.88) (Figure 2B). The large standard deviations found in these subgroup analyses again support a high degree of interpatient variability.

**Figure 3:**
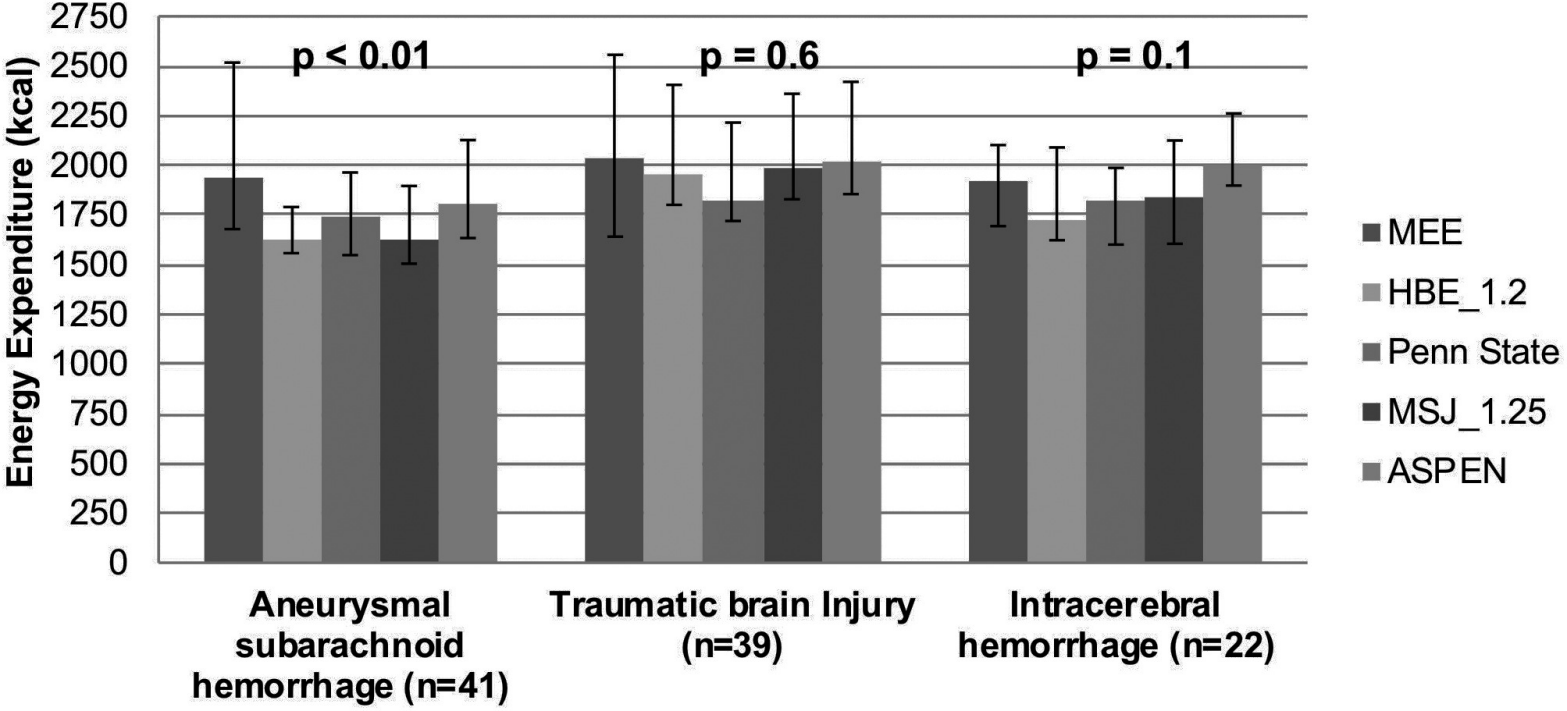
Energy expenditure by Brain injury type for patients with BMI < 30 kg/m^2^.

A further subgroup analysis was performed to determine differences between MEE and PEE for the most prevalent brain injuries, aSAH, ICH, and TBI. In the SAH subgroup, the median MEE as measured by IC was 1,929 (1,679–2,517) kcal compared to the PEE of 1,628 (1558–1790), 1,742 (1548–1964), 1,630 (1505–1896), and 1,811 (1634–2127) kcals as predicted by the HBE, Penn State, MSJ, and ASPEN equations, respectively (*p* < 0.01) (Table 4, Figure 3). In patients with aSAH, MEE was higher than PEE. No significant differences were found among MEE and PEE for ICH and TBI (*p* = 0.6 and *p* = 0.1, respectively).

**Table 4:** Energy expenditure for patients with BMI < 30 kg/m^2^ per brain injury.

	**MEE [median (IQR)] kcals**	HBE*1.2 [median (IQR)] kcals	**Penn state [median (IQR)] kcals**	MSJ*1.25 [median (IQR)] kcals	**ASPEN_avg BMI < 30 kg/m^**2**^ [median (IQR)] kcals**	***P*-value**
aSAH (*n* = 28)	1,929 (1,679–2,517)	1,628 (1,558–1,790)	1,742 (1,548–1,964)	1,630 (1,505–1,896)	1,811 (1,634–2,127)	< 0.01
ICH (*n* = 25)	1,926 (1,694–2,102)	1,732 (1,623–2,090)	1,822 (1,601–1,987)	1,837 (1,605–2,125)	2,010 (1,898–2,261)	0.1
TBI (*n* = 20)	2,043 (1,641–2,556)	1,955 (1,801–2,405)	1,828 (1,720–2,215)	1,991 (1,829–2,360)	2,019 (1,855–2,420)	0.6

## Discussion

Currently, this is one of the largest studies comparing MEE as measured by IC and PEE as measured by the HBE, Penn State, MSJ and ASPEN equations in an acute brain injury population. When comparing MEE vs. PEE in all patients with acute brain injury, no significant difference (*p* = 0.1) was found among measured or PEEs. This suggests there is no difference in MEE and PEE, and any equation could be used to accurately estimate REE in this patient population. This requires further interpretation, however, due to the high degree of inter- and intrapatient variability associated with the mean REEs. For example, the SD associated with HBE (Table 3) accounts for 19.4% of the average REE predicted by the equation. Furthermore, the Pearson's correlation calculated for HBE compared to MEE (Figure 1A) resulted in a poor correlation value (*r* = 0.372), also suggesting a weak association. These findings are consistent among all predicative equations used in this study. The highest degree of variability was found in the MEE, with SDs ranging from 23.9 to 29.2% of the average MEE. These data again highlight interpatient variability that exists in REE calculations and suggests that IC is capable of measuring REE more accurately than predictive equations.

Esper and colleagues found that predictive equations underestimated the energy requirements in a small cohort (n = 14) of intracranial hemorrhage patients compared to a controlled cohort of six severe TBI patients (18). Frankenfield et al. then investigated this in a larger cohort (*n* = 130) comparing the accuracy of PEE (calculated using one of two modified Penn State equations, depending on BMI and age) to MEE, as measured by IC, for TBI, acute ischemic stroke (AIS), and ICH patients. The authors concluded that the modified PSU equation was accurate 72% of the time in predicting REE (19). For critically ill elderly patients admitted to an ICU, Segadilha et al. found that IC is the preferred method of measuring REE compared to predictive equations (20). The results of our study support the conclusions of previous studies, identifying the poor accuracy rates of predicative equations when compared to MEE by IC (18–22).

Patients were stratified into categories based on BMI to perform additional subgroup analyses. A significant difference between MEE and PEE was found among the BMI < 30 kg/m^2^ group but not the BMI ≥ 30 kg/m^2^ group. The lack of a significant difference among patients with a BMI ≥ 30 kg/m^2^ again suggests any predictive equation could be used in lieu of IC; however, the same issue regarding variability persists and warrants use of IC to obtain an appropriate REE measurement.

There are other issues in addition to the difficulty of appropriately assessing REE in critically ill obese patients. According to Kee et al., critically ill obese patients with a BMI > 30 kg/m^2^, compared to those with a BMI < 30 kg/m^2^, have an odds ratio of 1.5 for experiencing malnutrition, due to clinician's misinterpretation of obese individuals having reservoirs of nutrition secondary to high BMIs (23). Critically ill obese patients are also less capable of mobilizing fat stores than their lean counterparts, as shown by Jeevanandam et al. in obese trauma patients (24). Combined, these factors put critically ill obese patients at an increased risk of malnutrition. The results of this subgroup analysis underline the importance of obtaining accurate REE in these patients and further support the use of IC whenever possible, as recommended in ASPEN guidelines and literature reviews of critically ill patients (7, 25, 26).

Patients with acute brain injury experience fluctuating metabolic states, making it difficult to estimate accurate REE (1, 18). In addition to analyzing patients based on BMI, a subgroup of these patients was analyzed based on type of acute brain injury, specifically aSAH, ICH, and TBI. Of the acute brain injuries analyzed, only the aSAH group had a significant difference found when comparing PEE and MEE (*p* < 0.01). The predictive equations in aSAH patients appeared to underestimate the metabolic needs of the patients compared to MEE. Based on the significant difference found in this analysis and the consistent underestimation, these results suggest predictive equations are inaccurate when compared to IC measuring REE for patients with aSAH. This could put these patients at an increased risk of hospital-acquired malnutrition if predicative equations are used. It's also unclear how complications occurring within patients with aSAH, such as fever, impact REE. Twelve of the aSAH patients in this study (30%) experienced a temperature > 38°C within 24 h prior to IC. As these patient populations experience changes in metabolic function, performing IC may also reveal useful metabolic information that might be translated into clinical information and have the potential to serve as both prognostic indicators or treatment targets (27).

Furthermore, the predictive equations appeared to underestimate REE in the ICH and TBI group, with the exception of the ASPEN equation overestimating in ICH patients; however, there were no significant differences found among these subgroups so conclusions cannot be made. The lack of significant difference suggests that any equation can be used to predict REE in patients with ICH or TBI. Given the wide IQRs associated with each median value, there is again evidence of high degrees of variability. This high degree of variability could translate to under- or overfeeding patients with ICH or TBI. This is consistent with what was found in the previous analyses in this study, reinforcing the recommendation to use IC whenever possible.

This study was able to capture a large number of patients with a variety of acute brain injuries. Patient demographics and characteristics of this study are similar to previous studies, especially considering age, BMI, and ICU status (16, 18, 28). The predictive equations used in this study are commonly found in the literature and in practice, even when IC is available (1, 7, 16, 18, 28). This study found that when comparing means of MEE and PEE, no significant differences are found; however, when these averages are assessed individually, there is a high degree of interpatient variability.

One limitation to this study is that we did not look at nutritional outcomes of these patients as it was not within the scope and design of this study. Certain patient characteristics such as any active renal replacement therapy, high FiO_2_ requirements, chest tubes requiring suction, or clinical instability preventing temporary disconnection from the ventilation circuit may be limitations to performing IC in a timely manner and may contribute to a delay in obtaining IC earlier in the hospital. An additional limitation is that we did not analyze the quantitative effects of comorbidities, sedation, or infection on REE or patient outcomes.

Future studies should focus on outcomes of patients using PEE via predictive equations vs. MEE by IC, which would allow for cost analyses and potentially reinforce the findings of this study, encouraging the use of IC when feasible. Additionally, it would be useful to further investigate whether IC findings can be used as a surrogate marker for other prognostic indicators or treatment targets and to determine if disease severity has an impact on REE.

## Conclusion

This study demonstrates that the predictive equations assessed in this study may calculate, on average, similar REEs as IC measures; however, the high degree of variability that exists among REE discovered in this study emphasizes the likelihood of under- or overfeeding acute brain injured patients when using predictive equations to estimate REE. In accordance with ASPEN 2016 guidelines, this study supports the use of IC over predictive equations in order to obtain the most accurate measurement of a patient's REE and minimize the likelihood of under- or overfeeding patients with acute brain injury.

## Data Availability Statement

The datasets generated for this study are available on request to the corresponding author.

## Ethics Statement

The studies involving human participants were reviewed and approved by University of North Carolina at Chapel Hill Institutional Review Board. Written informed consent for participation was not required for this study in accordance with the national legislation and the institutional requirements.

## Author Contributions

KM, JJ, and DR conceived of and designed the analysis, performed data analysis, reviewed and edited the draft, and final manuscript. WW, AC, and AD performed data analysis, wrote the draft of the manuscript, and approved the final manuscript. KD and EB conceived of the analysis, reviewed the manuscript, and approved the final manuscript.

### Conflict of Interest

The authors declare that the research was conducted in the absence of any commercial or financial relationships that could be construed as a potential conflict of interest.
